# Short-chain fatty acids—a key link between the gut microbiome and T-lymphocytes in neonates?

**DOI:** 10.1038/s41390-025-04075-0

**Published:** 2025-04-30

**Authors:** Tram N. Y. Bui, Ayamita Paul, Shalini Guleria, Justin M. O’Sullivan, Gergely Toldi

**Affiliations:** Liggins Institute, The University of Auckland, Auckland, New Zealand

## Abstract

**Abstract:**

Infancy is a vulnerable and critical phase in the acquisition of the gut microbiome and the establishment of immune function. Short-chain fatty acids (SCFAs), such as acetate, propionate and butyrate, are compounds mostly produced by the microbiome through various metabolic pathways and play an indispensable role in connecting the microbiome and the adaptive immune system. This review aims to summarise recent findings regarding the intricate relationship between SCFAs, the gut microbiome, and T lymphocytes with a focus on early life interactions. The paper discusses factors affecting the establishment of the neonatal microbiome, especially human milk versus formula milk, and how these influence SCFA concentrations in feces, which in turn directly impact T cell development and function. Despite recent advances in understanding the role of gut microbiome derived SCFAs in adults, a significant knowledge gap remains in translating these findings to neonates and exploring the utility of SCFAs as a potential therapeutic intervention in inflammatory complications of preterm and term neonates.

**Impact:**

- This review highlights potential therapeutic applications of short-chain fatty acids (SCFAs) in neonatal care, particularly in preventing and treating inflammatory conditions. This could lead to new treatment strategies for conditions like NEC and other immune-mediated disorders in neonates.
- By identifying significant knowledge gaps in neonatal SCFA research, this review helps future investigations toward understanding SCFA mechanisms specifically in neonates, potentially leading to age-appropriate therapeutic interventions.
- Understanding the relationship between early-life factors (such as feeding methods and microbiome development) and immune system development through SCFAs could inform public health policies and recommendations for infant nutrition and care practices.

## Introduction

The human gut microbiome plays a central role in maintaining health, influencing major biological functions such as metabolism and immunity. The establishment of the microbiome starts very early and is determined by many factors such as the mode of delivery, diet or exposure to antibiotics.^1^ Importantly, the gut microbiome of neonates is distinctively lower in diversity than that of adults.^2^ Early life exposure to the gut microbiome and its metabolites, such as Short-chain fatty acids (SCFAs), can have long-lasting effects on immune health, and may be a crucial element in the prevention of allergies and autoimmune disease.^3^ SCFAs, including acetate, propionate, and butyrate, are produced by the gut microbiome during the fermentation of dietary fibers and, in the case of neonates, mostly human milk oligosaccharides (HMOs), which have shown to possess various health benefits, including anti-inflammatory properties.^4^ Moreover, the crosstalk between the gut microbiome, SCFAs, and T cells is thought to play a key role in the maintenance of immune responses. This is of particular interest in early life, during the period when the activity and memory of the adaptive immune system are still maturing.^5^ However, our knowledge of this complex interplay and its impact on early life is still in its infancy. In this review, we discuss current advances in this field, emphasizing how the gut microbiome, SCFAs, and T cells interact and influence each other in early life.

A comprehensive literature search was conducted using PubMed, Medline, and Google Scholar databases, focusing on studies published until May 2024. Keywords included “SCFAs”, “microbiome”, “T cells”, “feces”, and “neonate”. Studies selected for review were restricted to original research, review articles, and pertinent clinical studies that clearly addressed the role of SCFAs in immune priming and intestinal epithelial health. Data extracted encompassed experimental models, T cell mechanisms, molecular signaling pathways, and therapeutic interventions. The literature’s thematic synthesis emphasized recurring findings and potential translational significance to human health.^6^

## Neonatal gut microbiome

The neonatal gut microbiome is a complex ecosystem of microorganisms. The earliest bacterial species of the gut microbiome originate from the mother through swallowed amniotic fluid during the fetal period and develop dynamically through delivery, milk intake, contact with the mother’s skin, and environmental exposure.^2,7^ These factors significantly influence the diversity and composition of the gut microbiome during the 1st years of life.^8,9^

Preterm neonates, who are often born by C-section, have higher antibiotic exposure, formula feeding, and experience prolonged hospitalization, exhibit distinct differences in microbiome progression compared to healthy neonates.^10–12^ In particular, vaginally delivered neonates typically harbor bacteria that resemble those found in the maternal vaginal microbiome, leading to the presence of potentially beneficial microbes (e.g., *Lactobacillus*, *Bifidobacterium*, and *Bacteroides* spp.).^13,14^ By contrast, those delivered by C-section have different bacterial profiles with significantly lower proportions of specific genera, such as *Bacteroides*, *Parabacteroides*, and *Clostridium*.^15–18^ Another study showed that vaginally born preterm neonates, exclusively breastfed and not exposed to antibiotics by 10 days postnatally also had fewer Firmicutes and more Proteobacteria, compared to neonates born at term.^19^ Their gut microbiome is characterized by lower diversity and abundant opportunistic pathogens such as Proteobacteria and *Enterobacteriaceae* spp.^10,12,20^

Additionally, perinatal and postnatal antibiotic use in preterm is associated with a significant reduction in *Bifidobacterium* and *Lactobacillus* spp.^1,11^ This can lead to persistent dysbiosis and an increased risk of infectious diseases such as necrotizing enterocolitis (NEC), and chronic lung disease.^21^ Specific bacteria, such as *Escherichia coli* and *Klebsiella* spp., have been linked to altered brain microstructure and lead to neurodevelopmental impairments in preterm neonates.^22^ These differences in the gut microbiome of preterm neonates might lead to lower SCFA production compared to the healthy ones.

## Short-chain fatty acid production by the microbiome

SCFAs are small ubiquitous molecules. These organic acids have fewer than six carbon atoms in their hydrocarbon chain, connected with a carboxylic acid functional group (COOH). The most abundant types of SCFA found in humans are acetate with two, propionate with three, and butyrate with four carbon atoms.^23^ In neonates, SCFAs are produced within their intestinal lumen by anaerobic bacterial fermentation of mainly undigested dietary carbohydrates, and to a lesser extent by proteins from milk, and then further converted into acetate, propionate, and butyrate through various pathways such as the bifid shunt, acetyl-CoA, acrylate, and succinate.^24–26^

The SCFA concentrations in neonates are initially low but increase exponentially with the establishment of the gut microbiome. In the 1st months after birth, luminal oxygen levels in the neonatal gut decrease.^9^ This allows for the colonization of some obligate anaerobes like *Bacteroidaceae*, *Lachnospiraceae*, and *Ruminococcaceae*, the most abundant bacteria in adults, making the gut considered mature and “adult-like”, thus the SCFA concentrations also increase.^9^

There are several bacterial genera, which produce SCFAs in the human gut microbiome, are shown in Table 1. Almost all belong to Actinomycetota, Bacteroidetes, Firmicutes, and Proteobacteria phyla. Gram-negative bacteria generally produce acetate and propionate, while Gram-positives produce butyrate. Acetate is produced by a broad range of bacteria in the human gut, making it the most abundant SCFA in the human body and environment.^27^ The early establishment of a diverse gut microbiome is essential for optimal SCFA production.^28^

**Table 1:** Short-chain fatty acid producers in the gut microbiome

Phylum	Class	Order	Family		Genus	A	B	P	References
Actinomycetota	Actinomycetia	Bifidobacteriales	Bifidobacteriaceae	Bifidobacterium		x	x		^86–88^
		Propionibacteriales	Propionibacteriaceae	Propionibacterium		x		x	
				Acidipropionibacterium		x		x	
Bacteroidetes	Bacteroidia	Bacteroidales	Prevotellaceae	Prevotella		x		x	^86,89–91^
			Bacteroidaceae	Bacteroides		x	x	x	
			Porphyromonoadaceae	Porphyromonas		x	x	x	
Firmicutes	Bacilli	Lactobacillales	Lactobacillaceae	Lactobacillus		x		x	^92–103^
		Bacillales				x	x	x	
	Clostridia	Clostridiales	Clostridiaceae	Clostridium		x	x		
				Intestinimonas		x	x		
			Peptococcaceae	Pelotomaculum				x	
		Eubacteriales	Ruminococcaceae	Faecalibacterium			x		
				Ruminococcus			x	x	
			Lachnospiraceae	Anaerostipes		x	x	x	
				Anaerotignum		x	x	x	
				Anaerobutyricum			x		
				Blautia		x	x	x	
				Butyrivibrio			x		
				Coprococcus		x	x	x	
				Roseburia			x	x	
			Eubacteriaceae	Acetobacterium		x	x	x	
				Eubacterium		x	x	x	
			Lactobacteriaceae	Butyribacterium		x	x		
	Negativicutes	Selenomonadales	Selenomonadaceae	Selenomonas		x		x	
		Veillonellales	Veillonellaceae	Megasphaera		x	x	x	
				Veillonella		x		x	
Proteobacteria	Alphaproteobacteria	Rhodospirillales	Rhodospirillaceae			x	x	x	^52,96,103–106^
	Deltaproteobacteria	Syntrophobacterales	Syntrophaceae	Smithella		x	x		
	Gammaproteobacteria	Enterobacterales	Enterobacteriaceae	Escherichia			x	x	
				Enterobacter		x	x	x	
		Pseudomonadales	Pseudomonadaceae	Pseudomonas		x	x	x	
Verrucomicrobiota	Verrucomicrobiae	Verrucomicrobiales	Akkermansiaceae	Akkermansia		x		x	^104^
Fibrobacterota	Fibrobacteria	Fibrobacterales	Fibrobacteraceae	Fibrobacter				x	^107^
Fusobacteriota	Fusobacteriia	Fusobacteriales	Fusobacteriaceae	Fusobacterium		x	x	x	^108^

*A* acetate, *B* butyrate, *P* propionate.

## Short-chain fatty acid concentrations in feces

SCFAs are produced in the gut and absorbed by intestinal epithelial cells (IECs), enter the portal vein, and are transported to the liver, influencing metabolic processes such as gluconeogenesis and lipogenesis.^29^ Thus, SCFA concentrations are significantly higher in the large intestine, particularly in the colon, compared to portal and peripheral venous blood.^30^ Therefore, fecal SCFA concentrations in neonates is mainly reported. These numbers fluctuate depending on the differences in gut microbiome composition, and feeding methods. Table 2 shows the SCFA concentrations in healthy individuals’ feces, particularly in meconium, 1-month-old neonates, and adults.^31,32^ In these studies, total fecal SCFAs in meconium were 119.95 µmol/g (16.5–740.77) and increased in 1-month-old neonates to 267.57 (67.32–649.06) µmol/g^31^ while the average concentration was ~543.4 µmol/g in adults.^32^ Acetate was the most predominant SCFA, followed by propionate and butyrate. These proportions were comparable at 1 month of age but differed significantly in adults.

**Table 2:** The concentrations of short-chain fatty acids (SCFAs) (µmol/g) in healthy neonates and adults. Pooled data adapted from^31,32^

SCFA	Meconium (*n* = 20)	1 month old (*n* = 41)	Adult (*n* = 50)
Acetate	88.49 (12.42–393.51)	182.21 (55.12–466.02)	209.7 (21.5–400.5)
Butyrate	8.96 (1.19–191.3)	22.95 (2.18–313.87)	176.0 (45.2–503.3)
Propionate	13.93 (1.57–234.50)	32.87 (5.07–106.6)	93.3 (33.8–185.1)

On the other hand, fecal SCFA concentrations in preterm neonates are significantly lower than term babies because of lower bacteria populations, as discussed in Part II. Westerbeek and colleagues found the concentration of acetate, butyrate, and propionate in preterm infants’ meconium were 97.8 (62.9–99.2) µmol/g, 0.3 (0.1–19.6) µmol/g, and 0.7 (0.3–32.7) µmol/g respectively. The fecal SCFAs increased when these babies were at 1 month old to 75.6 (45.9–99.0) µmol/g, 0.5 (0.0–26.0) µmol/g, and 17.0 (0.2–44.9) µmol/g in acetate, butyrate, and propionate respectively.^33^ Moreover, total fecal SCFA concentrations in extremely preterm neonates are also significantly lower than in those born less prematurely.^34^ Besides, perinatal antibiotic use, such as intrapartum prophylaxis, affected the microbiome establishment in preterm neonates, reducing SCFA concentrations by 80% compared to full-term neonates at 2 days of age.^11^ There are very limited studies explored SCFAs concentration in neonatal plasma. Further research is needed to investigate SCFA levels in neonates, the relationship between plasma SCFA and fecal SCFA concentrations at different time points, and the impact of the differences in the gut microbiome composition.

In addition, a recent study suggested that the concentrations of propionate and butyrate are significantly higher in the cervicovaginal fluid of pregnant women preceding a preterm delivery compared to those who delivered at term. This raises the possibility that SCFAs could be used as potential biomarkers for predicting preterm birth.^35^

## Differences in short-chain fatty acid concentrations between breastfed and formula-fed neonates

SCFAs are produced differently depending on the type of milk consumption.^36^ There is a systematic review study showed that all three types of SCFAs increase significantly from birth until 6 months in breastfed and formula-fed neonates.^36^ However, the differences in the proportion of SCFAs depend on the feeding methods.^36^ Up to 6 months of age, propionate, butyrate, and total SCFA concentrations are higher, but acetate is lower in formula-fed compared to breastfed neonates. In breastfed neonates, the *Bifidobacteriaceae* family is enriched because they express the genes that utilize fucosylated HMOs (2ʹfucosyllactose and difucosyllactose) and non-fucosylated HMOs (lacto-N-hexose) to produce acetate.^37–39^ Luyao and colleagues also observed an increase in the number of *Bifidobacteria* spp. corresponding to the increase in acetate concentration in the feces.^40^ Fukudo and colleagues showed that acetate, produced by *Bifidobacteria* spp., improved intestinal defenses and protect against *E. coli* in mice.^41^

Additionally, the effect of human milk on gut bacteria may be related to large amounts of lactoferrin, which limits the availability of iron necessary for bacteria to carry out enzymatic reactions and regulate gene expression.^42,43^ *Bifidobacteria* spp. are well adapted to conditions with low iron content and lowering iron availability results in decreased butyrate production. These reasons could be used to explain the high concentration of acetate in breastfed neonates. Discontinuation of breastfeeding may result in a decrease in lactoferrin and an increase in free iron, thus leading to increase intestinal butyrate production.^44^

Moreover, *Bifidobacterium* spp. also interacts with species in Bacteroides family to modulate SCFA production. Abundance of Bacteroides in breastfed infants significantly impacts propionate concentration in their gut. Bacteroides are major producers of SCFAs, which play crucial roles in gut health and systemic inflammation, influence the gut microbial ecology, and reduce the plenty of pathogenic bacteria like *Escherichia coli*.^45^

On the other hand, the higher propionate and butyrate concentrations observed in formula-fed neonates may result from the greater bacterial diversity observed in their gut compared to exclusively breastfed neonates.^46^ This diversity may come from the environment and the differences in the composition or absorption of nutrients in formula milk compared to human milk in the neonatal gut.^26,47^ Higher concentrations of branched-chain fatty acids derived from amino acid metabolism found in formula-fed neonates are linked to the higher protein content of formula than human milk.^26^ The availability of these substrates is likely to increase the abundance of proteolytic bacteria, such as *Bacteroides* and *Clostridia* genera, seen in formula-fed neonates, leading to higher concentrations of propionate and butyrate.^26^

Furthermore, some studies have demonstrated that the application of several species of *Lactobacilli* and *Bifidobacteria* spp. as a probiotic supplementation in formula milk helps to increase acetate levels in feces and modulates gut microbiome perturbations, leading to reduce inflammation in both full-term and preterm neonates.^48^ Nevertheless, follow-up studies would be required to determine the direct effects on these children’s health outcomes in the long term.

## Neonatal T cells

T cells, or T lymphocytes, are essential components of the adaptive immune system and play a central role in antigen-specific responses of the immune system. These cells are characterized by the presence of T cell receptors (TCRs) on their surface, which enable them to recognize antigens presented by major histocompatibility complex (MHC) molecules on antigen-presenting cells. T cells can be broadly categorized into CD4^+^ T cells, also known as helper T cells, and CD8^+^ T cells, also called cytotoxic T cells.^49^

Neonatal T cells are different from adult T cells. Neonatal T cells represent a dynamic population poised to rapidly differentiate into regulatory or effector cells based on the host’s requirements.^5^ These neonatal T cells exhibit unique characteristics, such as reduced interleukin-4 (IL-4) and interferon-gamma (IFN-g) production, which is associated with the absence of a memory T cell population abundance in adults.^50^ In addition, neonatal CD4^+^ T cells have reduced TCR signaling responses compared to adult cells.^51^ Additionally, these T cells have distinct responses to stimuli,^52^ with differences in proliferation rates when stimulated with neonatal versus adult dendritic cells.^53^ Besides, some studies have indicated that neonatal T cells have a propensity to differentiate into short-lived effector cells rather than memory cells, displaying a distinct gene expression profile compared to adult T cells.^54^ Neonatal regulatory T cells (Tregs) expressing Foxp3 have suppressive functions similarly to their adult counterparts, and their proportion increases in peripheral blood in the first few weeks of life.^55–57^ Neonatal T cells also exhibit unique immune responses, with a high production of IL-8, a prevalent Th2 profile, and innate inflammatory responses, setting them apart from adult T cells. These differences in neonatal T cell functionality are further highlighted by their distinct responses to CD28 activation and requirements for exogenous IL-2 for proliferative responses.^58^

## Impact of gut microbiome derived short-chain fatty acids on neonatal T cells

SCFAs are considered mediators in the interaction between the gut microbiome and the immune system. They are efficiently absorbed by IECs through two protein transpoters which are monocarboxylate transporter 1 (MCT1) and sodium-coupled monocarboxylate transporter 1 (SMCT1).^59–61^ Beyond serving as energy sources for IECs, SCFAs act as signaling molecules influencing diverse cellular processes through the activation of specific G protein-coupled receptors (GPRs), primarily GPR41 (FFAR3) and GPR43 (FFAR2).^62^ GPR43 activation by SCFAs notably regulates neutrophil chemotaxis and T cell differentiation.^62^ Additionally, SCFAs also influence immune function via epigenetic mechanisms, primarily through histone deacetylase (HDAC) inhibition. This epigenetic regulation significantly changes gene expression, cytokine production, and T cell functions. For example, SCFAs promote anti-inflammatory cytokines such as IL-10 while suppressing pro-inflammatory cytokines, including TNF-α and IFN-γ, mediated through combined GPR signaling and HDAC inhibition pathways.^63,64^

Different types of SCFAs have different functions. For example, butyrate could inhibit the differentiation of pro-inflammatory Th17 cells while promoting the generation and function of Tregs, a crucial step in maintaining immune homeostasis.^65,66^ Acetate and propionate enhance Th17 cell formation and expand Th1 cell populations through IL-12 signaling pathways, essential for defense against pathogens and inflammatory responses.^65^ Propionate further influences T cell metabolism via phosphorylation of ribosomal protein S6, an mTOR signaling target, affecting cytokine expression such as IFN-γ, IL-10, and IL-17, and stimulating Tregs differentiation by enhancing Foxp3 expression.^67,68^

In addition, some studies found that butyrate prominently maintains mucosal barrier integrity and mitigates inflammation by promoting tight junction protein and mucin expression through HDAC inhibition and GPR109a signaling pathways.^69,70^ Moreover, butyrate also protects against NEC in neonatal animal models by inducing inhibitory proteins such as single immunoglobulin IL-1-related receptor and toll-interacting protein, which modulate excessive toll-like receptor signaling.^71^ Thus, butyrate might exhibit notable therapeutic potential for neonatal gastrointestinal diseases by reinforcing barrier function and reducing inflammation.^72^ Other studies investigating the impacts of SCFAs on T cell subset modulation in human adults are presented in Table 3.

**Table 3:** Impact of SCFAs on T cell subset modulation in human adults

SCFA	T cell subset	Mechanism	Observed Effects	Ref.
Butyrate	Tregs	Increased IL-10 production	Increased suppressive capacity of Tregs	^109^
Butyrate	Tregs	Increased IL-10 and decreased IL-6 production	Enhanced induction of Tregs	^110^
Butyrate, Propionate	CD8^+^ T cells	Suppression of IL-12 production	Inhibited antigen-specific T cell activation	^111^
Acetate	Memory CD8^+^ T cells	Modulation of acetate-assimilating enzymes, glutaminase activation, TCR-triggered calcium flux suppression	Enhanced IFN-γ production, improved pathogen clearance	^112^
Acetate, Butyrate, Propionate	CD4^+^ T cells and ILCs	Aryl hydrocarbon receptor (AhR) and hypoxia-inducible factor (HIF)1α pathways	Increased IL-22 production	^113^
Butyrate	CD4^+^ T cells	HDAC inhibition, GPR43 signaling	Inhibited activation, proliferation, and cytokine production	^114^
Butyrate	CD8^+^ T cells, CAR T cells	mTOR activation, HDAC inhibition	Enhanced anti-tumor activity	^81^

There are a minimal number of studies in neonates showing the impacts of SCFAs in T lymphocytes. A study using cord blood cells indicated that propionate and butyrate increased IL-4 expression when naïve CD8^+^ T cells were activated in the presence of IL-6 and led to the differentiation of CD8^+^ T cells into non-classic TC2 cells.^73^ This change occurs alongside a shift from sugar-based to fat-based energy production in the cells. These effects involve several cellular mechanisms, including GPR signaling, HDAC inhibition, and processes that depend on caspase activity. Another study on 12 healthy term neonates indicated that exposure to butyrate and propionate in naïve non-Treg CD4^+^ cells could enhance in vitro induced Tregs (iTreg) differentiation and function by increasing histone H4 acetylation at Treg loci, and led to increase suppressive capacity against responder T cells.^74^

**Fig. 1:**
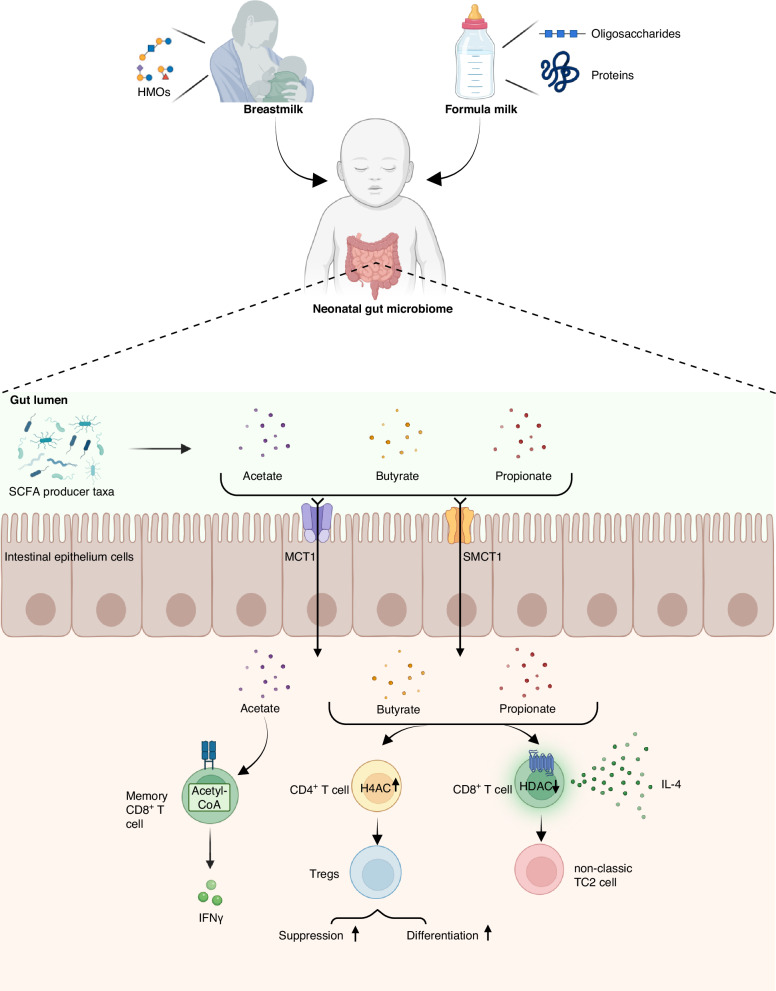
Impact of gut microbiome derived short-chain fatty acids (SCFAs) on neonatal T cells. Nutrients in breastmilk and formula milk shape the gut microbiome composition in neonates, promoting SCFA production (acetate, butyrate, propionate). After crossing intestinal epithelial cells (IECs) via MCT1 and SMCT1 protein transporters, acetate enhances memory CD8+ T cell activity by modulating acetate-assimilating enzymes, increasing IFN-γ production. Butyrate and propionate promote naïve CD4+ T cell differentiation into regulatory T cells (Tregs) and enhance their suppressive function through increased histone H4 acetylation (H4AC). Additionally, butyrate and propionate reduce histone deacetylase (HDAC) activity and thus elevate IL-4 expression, driving differentiation of naïve CD8+ T cells into non-classic cytotoxic T (TC2) cells.^26,59–61,73,74^

In addition, Toldi and colleagues assessed 38 term neonates born by C-section at two time points, at birth and 3 weeks of age. They found that the proportion of Tregs increases nearly two fold higher in exclusively breastfed neonates compared with those who received formula milk only. These breastfed neonates show a specific and Treg-dependent reduction in proliferative T cell responses to non-inherited maternal antigens associated with reduced in inflammatory cytokine production. They also observed the enrichment of SCFA-producing taxa in feces of exclusively breastfed neonates.^57^ While these laboratory studies provide important insights into how SCFAs influence neonatal T cell development, more clinical research is needed to understand how these effects occur in neonates and what they mean for long-term immune system development. Figure 1 summarizes the impact of gut microbiome derived SCFAs on neonatal T cells.

Clinical studies investigated the effects of oral SCFA supplements in adults, particularly butyrate, in treating inflammatory bowel diseases (IBD).^75^ In a randomized, double-blind, placebo-controlled trial involving patients with mild to moderate ulcerative colitis, the combination of oral mesalamine with 4 g butyrate daily for 6 weeks resulted in significant improvements compared to placebo.^76^ In the case of Crohn’s disease, the contribution of 4 g butyrate was divided into 2 doses for 8 weeks in a group of 13 patients. Butyrate was well tolerated, and after treatment, leukocyte blood count, erythrocyte sedimentation rate, mucosal levels of NFkB and IL-1b significantly decreased.^77^ Another study of 216 patients reported that treatment with 307 mg butyrate and 250 mg inulin prebiotic improved symptoms and mucosal appearance at endoscopy.^78^ This result provides evidence to support that prebiotics could be combined with SCFAs to enhance the therapeutic effect.

On the other hand, SCFAs were also shown to have potentially harmful effects on health. A study on mice showed that oral administration of 200 mM acetate for 6 weeks led to a 400% increase in acetate concentration in kidney tissues, which was associated with the development of chronic kidney disease, highlighting the disparity between physiological and harmful levels.^79^ Another study performed on samples from preterm neonates indicated that high concentrations of butyrate (150 mM) can induce significant intestinal epithelial injury resembling NEC. This injury predominantly occurs via necroptosis, and is particularly pronounced in premature intestinal cells due to their developmental vulnerability. Preterm newborns, whose intestinal mucosa is structurally and functionally immature, are especially susceptible to SCFA-induced injury, highlighting a developmental-stage dependency. Elevated butyrate levels increase oxidative stress and compromise mucosal integrity, particularly affecting stem cells and immature enterocytes located closer to the lumen. Furthermore, necroptosis inhibitors such as necrostatin-1 (Nec-1) have effectively reduced butyrate-induced cellular injury, suggesting that targeted intervention could mitigate SCFA-related adverse effects. Given this, caution is necessary when considering SCFAs’ potential impacts on immune priming in vulnerable populations, such as premature neonates, due to their susceptibility to necroptotic injury pathways.^80^

## Conclusion

The complex interplay between the gut microbiome, SCFAs, and T lymphocytes significantly influences early life immune development. SCFAs have demonstrated the ability to regulate T cell function and cytokine production, potentially modulating immune responses in various autoimmune and inflammatory disorders, including multiple sclerosis, rheumatoid arthritis, colitis, type 1 diabetes, IBD, cancer, and infectious diseases.^66,81–85^ Nevertheless, chronically elevated SCFA levels can lead to dysregulated T cell responses, resulting in tissue inflammation and disorders such as ureteritis and hydronephrosis.^79,80^ This emphasizes that while SCFAs hold beneficial therapeutic promise, their concentrations must be carefully regulated to avoid adverse outcomes.

Current knowledge predominantly stems from studies focusing on animal and adult T cells. Hence, significant gaps remain regarding the impact of SCFAs on neonatal T cells, particularly among preterm infants. Factors such as antibiotic exposure, prematurity, delivery mode, and variation in milk type profoundly alter neonatal gut microbiome composition, influencing immune health outcomes. Future research should prioritize elucidating these specific mechanisms in neonatal populations, paving the way for targeted interventions that harness SCFAs to optimize health outcomes from early life.

### Future directions of research

Several research avenues remain unexplored and require further investigation to deepen our understanding of the complex relationship between SCFAs, the microbiome and T lymphocytes, and its health implications. The following areas are considered important future research priorities to advance this field.

- The correlation between plasma and feces SCFA levels in term and preterm neonates
- The changes in plasma and feces SCFA levels throughout early life
- The impact of SCFAs on neonatal T cells in vitro and in vivo in comparison to adult T cells
- The impact of prematurity and inflammatory complications of preterm neonates, such as NEC, on SCFA levels
- The therapeutic use of SCFAs in preventing inflammatory complications in preterm and term neonates
- The impact of SCFA levels on infection outcomes in early life
- The impact of SCFA levels on the incidence of allergies and autoimmune diseases in later life
